# Activation of 4-1BB signaling in bone marrow stromal cells triggers bone loss via the p-38 MAPK-DKK1 axis in aged mice

**DOI:** 10.1038/s12276-021-00605-y

**Published:** 2021-04-15

**Authors:** Daqian Wan, Songtao Ai, Huoniu Ouyang, Liming Cheng

**Affiliations:** 1 Department of Orthopedics, Tongji Hospital affiliated with Tongji University School of Medicine, Shanghai, China; 2 Key Laboratory of Spine and Spinal Cord Injury Repair and Regeneration, Ministry of Education of the People’s Republic of China, Shanghai, China; 3 Department of Radiology, Shanghai Ninth People’s Hospital, Shanghai Jiaotong University School of Medicine, Shanghai, China; 4 Department of Neurosurgery, Shanghai Ninth People’s Hospital, Shanghai Jiaotong University School of Medicine, Shanghai, China

**Keywords:** Stem-cell differentiation, Mesenchymal stem cells, Ageing

## Abstract

Senile osteoporosis can cause bone fragility and increased fracture risks and has been one of the most prevalent and severe diseases affecting the elderly population. Bone formation depends on the proper osteogenic differentiation of bone marrow stromal cells (BMSCs) in the bone marrow microenvironment, which is generated by the functional relationship among different cell types in the bone marrow. With aging, bone marrow provides signals that repress osteogenesis. Finding the signals that oppose BMSC osteogenic differentiation from the bone marrow microenvironment and identifying the abnormal changes in BMSCs with aging are key to elucidating the mechanisms of senile osteoporosis. In a pilot experiment, we found that 4-1BBL and 4-1BB were more abundant in bone marrow from aged (18-month-old) mice than young (6-month-old) mice. Meanwhile, significant bone loss was observed in aged mice compared with young mice. However, very little data have been generated regarding whether high-level 4-1BB/4-1BBL in bone marrow was associated with bone loss in aged mice. In the current study, we found upregulation of 4-1BB in the BMSCs of aged mice, which resulted in the attenuation of the osteogenic differentiation potential of BMSCs from aged mice via the p38 MAPK-Dkk1 pathway. More importantly, bone loss of aged mice could be rescued through the blockade of 4-1BB signaling in vivo. Our study will benefit not only our understanding of the pathogenesis of age-related trabecular bone loss but also the search for new targets to treat senile osteoporosis.

## Bone formation: An off-switch for age-related osteoporosis

A signaling pathway that suppresses the production of bone-forming cells with increasing age could offer a useful target for the treatment of osteoporosis. Bone is replenished by osteoblasts, cells that originate from reservoirs of bone marrow stromal cells (BMSCs). In age-related osteoporosis, suppressive signals inhibit the formation of new osteoblasts. Researchers led by Huoniu Ouyang of Shanghai Ninth People’s Hospital and Liming Cheng at Tongji Hospital in China have identified an important component of this inhibition. They discovered that a signaling protein called 4-1BB is expressed at higher levels in BMSCs from aged mice than in those from young mice. Activation of this protein switches off osteoblast production and contributes to bone loss. Treatments that interfere with 4-1BB counter this degenerative process, suggesting a possible approach for therapeutic intervention.

## Introduction

Osteoporosis is defined as a deterioration of bone mass and microarchitecture with increasing fragility, which leads to fractures^1^. There are two main pathophysiological processes that can result in significant bone loss. The first is known as postmenopausal osteoporosis, which is derived from estrogen deprivation^2^. In contrast, a second type of osteoporosis, known as senile osteoporosis, most frequently affects older people, predisposing them to hip fractures^3^.

The skeleton is composed of two components: peripheral and axial. The peripheral (cortical) skeleton primarily contains compact plates (lamellae) organized around central nutrient canals. The axial skeleton is composed of trabecular (cancellous) bone. Trabecular bone is a honeycomb of vertical and horizontal struts in which bone marrow is located. Because bone marrow elements are the source of cells involved in bone turnover, the proximity to the cellular elements results in earlier and more sensitive responses by trabecular bone to whole-body changes in the bone remodeling rate^4^.

Bone turnover corresponds to the continuous cycle of destruction and renewal of bone as a consequence of the coupled action of osteoclasts and osteoblasts. Osteoclasts are derived from the monocytic hematopoietic lineage and share a common precursor with macrophages^5^. These cells are responsible for bone resorption and start the bone remodeling cycle^5^. In contrast, osteoblasts are fibroblastic-like cells that originate from bone marrow stromal cells (BMSCs) in the bone marrow. These cells have the capacity to generate a new osteoid and to complete its mineralization^6^. Increased bone resorption and/or decreased bone formation can cause bone loss. Investigating the mechanisms underlying bone formation and resorption is of importance to provide insights into senile osteoporosis.

BMSCs are located in specific bone marrow microenvironments that are responsible for the maintenance of stem cell populations, their controlled proliferation, and their differentiation into multiple lineages^7,8^. The bone marrow microenvironment, an extraordinarily heterogeneous and dynamic system, is generated by the functional relationship among different cells types found in the bone marrow via locally produced soluble factors that exert autocrine, paracrine, and endocrine activities^7,8^. Physiological bone marrow provides a suitable microenvironment for osteogenesis and the maintenance of bone homeostasis. In such conditions, BMSCs pass through a sequence of events ensuring proper osteoblast development^9^. However, with aging, the bone marrow microenvironment changes significantly, thus providing signals that repress osteogenesis^10^. Finding the signals that oppose BMSC osteogenic differentiation from the bone marrow microenvironment and identifying the abnormal changes in BMSCs in response to them with aging are key to elucidating the molecular mechanisms of senile osteoporosis.

To date, much has been learned about the basic biology of the TNF superfamily (TNFSF), their receptors (TNF receptor superfamily, TNFRSF), the intracellular signaling pathways activated by these receptors, as well as the roles of the TNFSF cytokines in a number of inflammatory and autoimmune diseases^11^. 4-1BB (also known as TNFRSF9) is an inducible stimulatory receptor mainly expressed on T cells and innate lymphoid cells; expression of its ligand 4-1BBL (also known as TNFSF9) is also inducible on APCs^12–14^. 4-1BB is currently being targeted with receptor agonists to promote antitumor T-cell responses in the context of clinical cancer immunotherapy^15^.

Previously, only a few studies have reported the role of 4-1BB in the skeletal system, and these mainly focused on its effect on osteoclast formation and function^16–18^. In a pilot experiment, we found that 4-1BBL and 4-1BB were more abundant in bone marrow from aged (18-month-old) mice than young (6-month-old) mice. Meanwhile, significant bone loss as a result of decreased bone formation and increased bone resorption was observed in aged mice compared with young mice. However, very little data have been generated with regard to whether high-level 4-1BB/4-1BBL in bone marrow was associated with bone loss in aged mice. To answer this question, we focused on the role of 4-1BB/4-1BBL signaling in BMSC osteogenic differentiation and osteoporosis pathophysiology. We found that 4-1BB was upregulated in BMSCs from aged mice, which resulted in the attenuation of osteogenic differentiation potential of BMSCs of aged mice via the p38 MAPK-Dkk1 signaling pathway. More importantly, trabecular bone loss of aged mice could be rescued through in vivo blockade of 4-1BB signaling. Our study will benefit not only our understanding of the pathogenesis of age-related bone loss but also the search for new therapy targets to treat senile osteoporosis.

## Materials and methods

### Animals

Male C57BL/6 mice (SIPPR-BK Laboratory Animal Co. Ltd, Shanghai, China) were housed under SPF conditions. All animal operations were approved by the Animal Ethics Committee of Tongji Hospital affiliated with Tongji University School of Medicine.

Each group included 10–12 animals. Animals were euthanized using isoflurane inhalation anesthesia followed by cervical dislocation. The right femur and #1 lumbar vertebra (L1) were prepared for micro-computed tomography (micro-CT) scan; the left femur and tibia were used for bone marrow cell isolation. Peripheral serum was taken for the measurement of propeptide type I procollagen (PINP) and tartrate-resistant acid phosphatase-5b (Trap-5b) (Immunodiagnostic Systems plc, Tyne and Wear, UK).

Animals were administered neutralization anti-4-1BB mAb (BioLegend, San Diego, CA, USA) intraperitoneally in 0.9% saline at a dose of 100 μg/animal weekly. After 8 weeks, animals were euthanatized for micro-CT and serum biomarker analysis.

### Micro-CT

Femurs and L1 vertebrae were harvested and preserved in 70% ethanol. Bones were scanned using a micro-CT instrument (SkyScan 1172; Bruker-microCT, Kontich, Belgium). Standard nomenclature and guidelines for assessment of bone microstructure were as recommended by the American Society for Bone and Mineral Research^19^. Scans for the cortical region were measured at the midpoint of each femur, with an isotropic pixel size of 21 μm and slice thickness of 21 μm, and used to calculate the average total cross-sectional area (mm^2^), bone area (mm^2^), and cortical bone thickness (Ct.Th). The trabecular bones of the distal femur and L1 vertebra were scanned at an energy level of 55 kVp, intensity of 145 μA, and a fixed threshold of 220. The trabecular bone volume fraction and microarchitecture were evaluated in the secondary spongiosa, starting proximately at 0.6 mm distal to the growth plate and extending 1.5 mm. Approximately 230 consecutive slices were made at the end of the growth plate and extending in a proximal direction, and 100 contiguous slices were selected for analysis. The main bone parameters were BV/TV (the relative volume of calcified tissue in the selected volume of interest), Tb.N (the trabecular number) and Tb.Sp (trabecular bone separation).

### Double calcein labeling

Mice were subcutaneously administered with calcein (Sigma) at a dose of 15 mg/kg at day 10 and day 3 before sacrifice. After fixation and embedding in polymethyl acrylate, femurs were serially cut into 50-μm-thick sections, and the double calcein labeling was observed with a fluorescence microscope (Nikon). The bone formation rate (BFR) was calculated as the inter-label width/labeling period with Image-Pro software.

### Histomorphometric analysis

Bone histomorphometry to quantify osteoclasts was performed in mouse femurs embedded in paraffin that were stained for Trap in a blinded and nonbiased manner. Osteoclasts were identified as multinucleated Trap-positive cells adjacent to bone. All measurements were confined to the secondary spongiosa and restricted to an area between 400 and 2000 μm distal to the growth plate-metaphyseal junction of the distal femur. The percentage of osteoclast surface (Oc.S) to bone surface (BS) was calculated.

### ELISA

4-1BB and 4-1BBL levels in bone marrow aspirates, peripheral blood serum, and culture media were measured with ELISA kits (RayBiotech, Norcross, GA, USA) according to the manufacturer’s instructions.

### Immunofluorescence labeling and sorting of bone marrow cells

MACS LS columns (Miltenyi Biotec) were employed to enrich bone marrow by either positive isolation of c-Kit+ cells by labeling with anti-c-Kit magnetic beads or negative depletion of lineage-negative cells by labeling with biotin-conjugated antibodies specific for lineage markers followed by anti-biotin magnetic beads. Cells were collected from the enriched bone marrow for further sorting. Cells were gated for flow cytometry or cell sorting as follows: Hematopoietic stem cell (HSC), Lin^-^Sca-1^+^c-Kit^hi^IL-7Rα^−^Flt3^−^Thy-1.2^+^; myeloid progenitor (MP), Lin^−^Sca-1^-^c-Kit^hi^IL-7Rα^−^.

### Isolation of bone marrow dendritic cells (DCs)

Bone marrow single-cell suspensions were cultured in complete medium (RPMI 1640 supplemented with 10% FBS, 2 mM L-glutamine, 100 U/ml penicillin, and 100 μg/ml streptomycin) supplemented with murine GM-CSF (10 ng/ml) and murine IL-4 (2 ng/ml) (R&D Systems, Minneapolis, MN). Fresh medium was added every 2 days. Nonadherent bone marrow DCs were harvested on day 6 for flow cytometry analysis.

### Flow cytometry

Cells were stained at 4 °C for 30 min with biotin-conjugated anti-4-1BB (17B5) and anti-4-1BBL (TKS-1, both from BioLegend) in PBS supplemented with 3% FBS and 0.02% azide. Then, the cells were washed and stained an additional 30 min at 4 °C with secondary antibody before flow cytometry analysis by a FACScan (BD Biosciences, Mountain View, CA).

### BMSC in vitro culture, osteogenesis induction, and treatment

The bone marrow from femurs and tibia was suspended in cold PBS and passed through a 70-μm filter. The supernatants were kept for use in ELISAs. Filtered bone marrow cells were suspended in PBS with 2% FBS and 0.1 g/L phenol red and then enriched for lineage negative (Lin−) cells using the SpinSep system (Stem Cell Technologies, Vancouver, BC, Canada). The cells were incubated with a murine progenitor enrichment cocktail (Stem Cell Technologies) on ice for 30 min, washed, and then incubated with dense particles on ice for 20 min. The cells were then centrifuged at 1200 × *g* for 10 min, and the cells at the density medium/PBS interface were collected.

Enriched BMSCs were seeded onto culture plates at a density of 0.1 × 10^6^ cells/cm^2^ in α-MEM containing 100 units/ml penicillin (Gibco BRL, Rockville, MD, USA) and 100 μg/ml streptomycin (Gibco). The medium was changed after 72 h, and adherent cells were maintained in culture with twice-weekly media changes.

To induce osteogenic differentiation, BMSCs were treated with 100 nM dexamethasone (Sigma Chemical Co., St. Louis, MO), 10 mM β-glycerophosphate disodium (Sigma), and 50 μg/ml ascorbic acid (Sigma).

Recombinant mouse 4-1BBL (His-tagged and cross-linked with anti-polyhistidine antibody; R&D Systems) was used at dose of 0.5 μg/ml to activate 4-1BB signaling in BMSC culture. Neutralization anti-4-1BB mAb (BioLegend) was used at dose of 5 μg/ml to block 4-1BB signaling in BMSC culture. Gallocyanine (Abcam, Cambridge, MA, USA) was used at dose of 50 mM to inhibit DKK1 activity. SB203580 (Cell Signaling Technology, Danvers, MA, USA) was used at dose of 10 μM to inhibit p38-MAPK activity.

### Alkaline phosphatase (ALP) and alizarin red staining

For ALP staining, BMSCs were washed with PBS and fixed with 4% paraformaldehyde for 10 min at 4 °C. Next, the cells were incubated in 0.1% naphthol AS-MX phosphate (Sigma) and 0.1% fast red violet LB salt (Sigma) in 2-amino-2-methyl-1,3-propanediol (Sigma) for 10 min at room temperature, washed with PBS, and then observed under a digital camera.

For alizarin red staining, BMSCs were washed with PBS and fixed with 4% paraformaldehyde for 10 min at 4 °C. After fixation, the cells were washed with PBS, incubated in 40 mM alizarin red (pH 4.2) for 30 min at 37 °C, washed with PBS and imaged.

### Quantitative RT-PCR

Total RNA was isolated from cultured cells using the RNeasy Mini Kit (Qiagen, Valencia, CA, USA) in accordance with the manufacturer’s protocol. Single-stranded cDNA was reverse transcribed from 1 μg of total RNA using reverse transcriptase with oligo-dT primers. Quantitative PCR analysis was performed on a 96-well plate ABI Prism 7500 Sequence Detection system (Applied Biosystems, Foster City, CA, USA) using SYBR Green PCR Master Mix (Takara Bio Inc., Otsu, Japan). The cycling conditions were as follows: 94 °C, 5 s; 60 °C, 34 s; and 72 °C, 45 s for 40 cycles. β-Actin was used as an internal control. The primer sequences are shown in Table S1.

### Western blotting

Cells were lysed on ice for 30 min in a buffer containing 50 mM Tris-HCl, pH 7.4, 150 mM NaCl, 1% Nonidet P-40, and 0.1% SDS supplemented with protease inhibitors (10 g/ml leupeptin, 10 g/ml pepstatin A, and 10 g/ml aprotinin). The proteins were separated by SDS-PAGE, transferred to a PVDF membrane, and detected using anti-4-1BB (Cell Signaling Technology), anti-Dkk1 (Thermo Fisher Scientific, Ann Arbor, MI, USA), anti-active β-catenin (Cell Signaling Technology), anti-phospho p38 MAPK (Cell Signaling Technology), anti-p38 MAPK (Cell Signaling Technology) and anti-β-actin (Cell Signaling Technology).

### Statistical analysis

Statistical significance was calculated by Student’s *t*-test for two-sample comparisons. ANOVA was applied for multiple comparisons with SPSS 16.0 software. Tukey’s test was used to find significant differences in ANOVA. Correlations of 4-1BB and 4-1BBL levels in serum with the trabecular bone volume fraction were calculated by Spearman correlation analyses. *p* < 0.05 was defined as significant. All the data were obtained from three independent experiments and are presented as the mean ± SEM unless otherwise specified.

## Results

### Bone loss in aged mice

The peak bone mass of mice is reached between 5 and 6 month of age^20^. Therefore, 6- and 18-month-old mice were used to investigate age-related bone loss in the current study. Femurs and L1 vertebrae were collected. Micro-CT was employed to assess the bone microstructure.

**Fig. 1:**
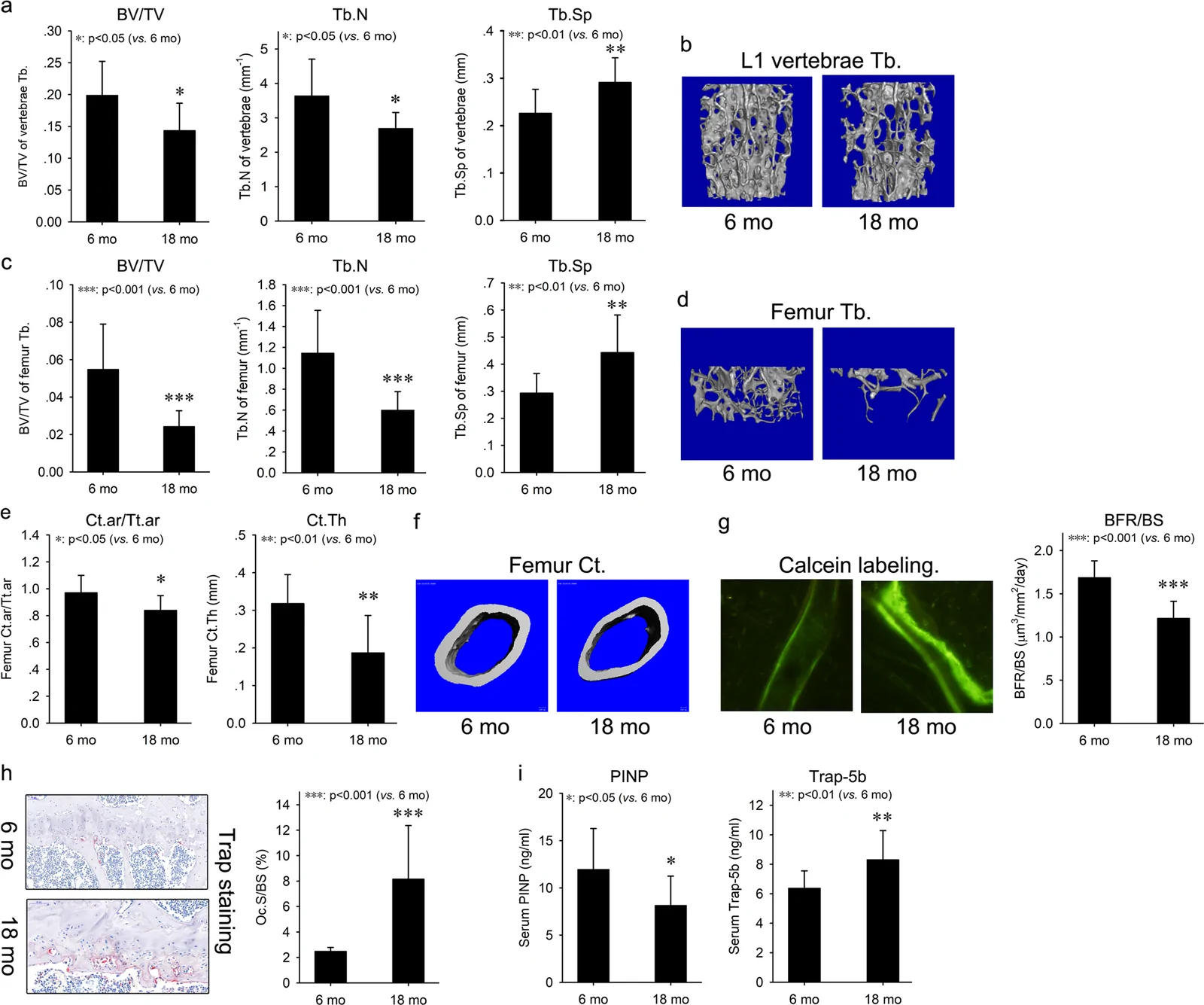
Bone loss in aged mice. **a** Bone volume fraction (BV/TV), trabecular bone number (Tb.N) and trabecular bone space (Tb.Sp) of the lumbar #1 (L1) vertebra from 6- and 18-month-old mice were determined by micro-CT. **b** Representative 3D reconstruction images of L1 vertebrae Tb. **c** BV/TV, Tb.N, and Tb.Sp of the distal femur. **d** Representative 3D reconstruction images of the distal femur Tb. **e** Ct.ar/Tt.ar and Ct.Th of cortical bone in the femur mid-shaft. **f** Representative 3D reconstruction images of femur Ct. **g** Double calcein labeling images and bone formation rate (BFR) quantification of 6- and 18-month-old mice. **h** Trap staining images and Oc.S/BS quantification of 6- and 18-month-old mice. **i** Serum levels of the bone turnover markers PINP and Trap-5b in 6- and 18-month-old mice were determined by ELISA. All the data were obtained from three independent experiments. Data are shown as the means ± SEMs. *p < 0.05, **p < 0.01, ***p < 0.001, 18 mo vs. 6 mo.

For trabecular bone, there was a 28% decrease in BV/TV, a 26% decrease in Tb.N and a 29% increase in Tb.Sp in the L1 vertebrae of 18-month-old mice compared with 6-month-old mice (Fig. 1a). There was a 56% decrease in BV/TV, a 48% decrease in Tb.N and a 51% increase in Tb.Sp in the distal femurs of 18-month-old mice compared with 6-month-old mice (Fig. 1c). For cortical bone, there was a 14% decrease in Ct.ar/Tt.ar and a 41% decrease in Ct.Th in the femur mid-shaft of 18-month-old mice compared with 6-month-old mice (Fig. 1e). 3D reconstruction images also showed the same pattern (Fig. 1b, d, and f).

In addition to micro-CT scanning, the double calcein labeling assay and histomorphometric analysis of Trap^+^ osteoclasts were performed to characterize in vivo bone formation and bone resorption activity in mice. In double calcein labeling assays, a 28% decrease in the BFR was observed in 18-month-old mice compared with 6-month-old mice **(**Fig. 1g**)**, suggesting the attenuation of new bone formation in aged mice. In histomorphometric analysis of Trap^+^ osteoclasts, an over 2-fold increase in Oc.S/BS was observed in 18-month-old mice compared with 6-month-old mice (Fig. 1h), suggesting the enhancement of bone resorption in aged mice.

Meanwhile, bone formation and resorption markers in peripheral blood serum were analyzed by ELISA to determine the bone turnover status of 6- and 18-month-old mice. A decrease in PINP (bone formation marker) and an increase in Trap-5b (bone resorption marker) were observed in serum from 18-month-old mice compared with 6-month-old mice (Fig. 1i).

### Accumulation of 4-1BB and 4-1BBL in the bone marrow microenvironment and upregulation of 4-1BB in BMSCs from aged mice

**Fig. 2:**
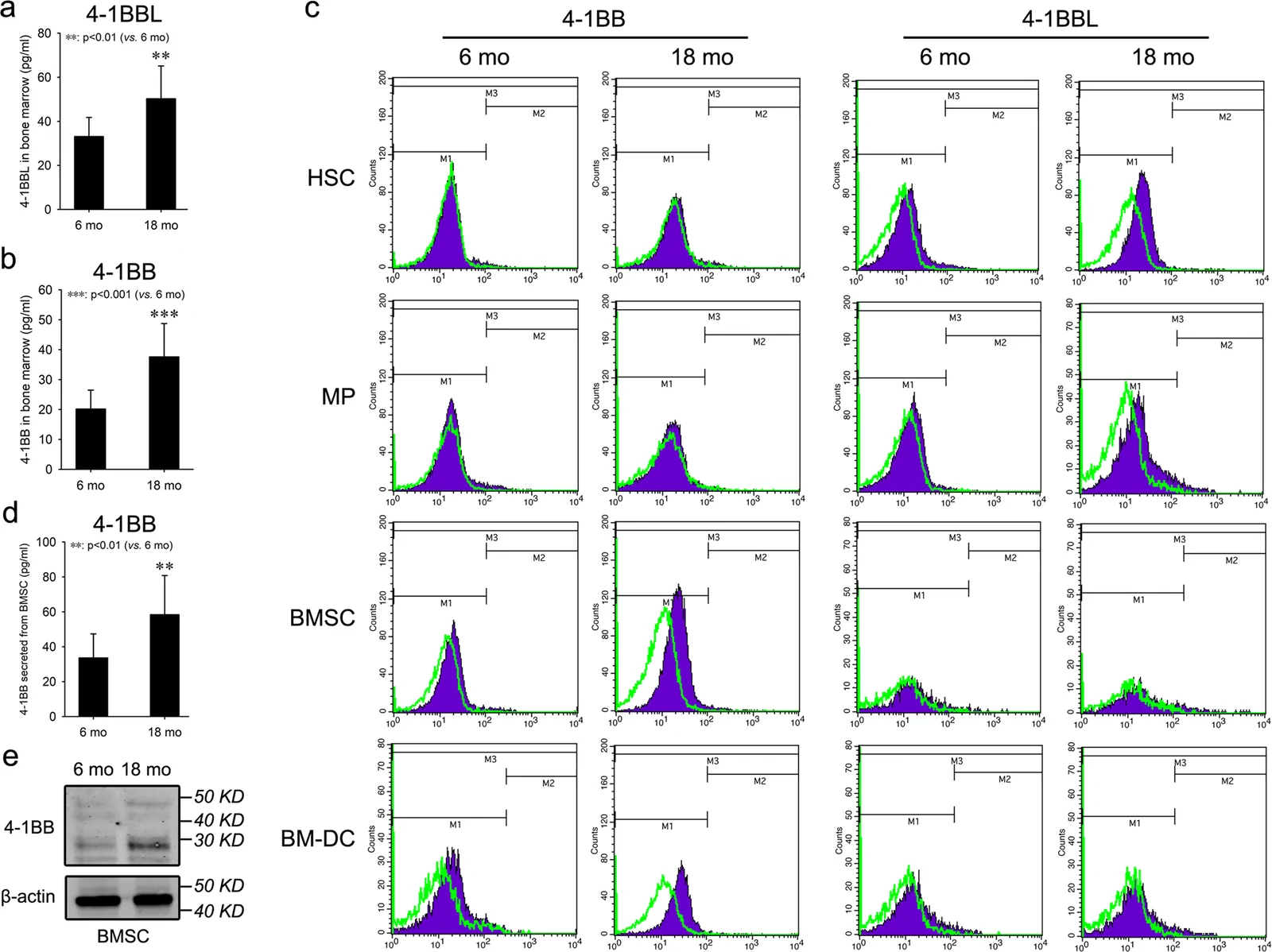
Accumulation of 4-1BB and 4-1BBL in the bone marrow microenvironment and upregulation of 4-1BB in BMSCs from aged mice. **a** 4-1BBL levels in bone marrow from 6- and 18-month-old mice were determined by ELISA. **b** 4-1BB levels in bone marrow from 6- and 18-month-old mice. **c** 4-1BB and 4-1BBL expression on hematopoietic stem cells (HSCs), myeloid progenitors (MPs), bone marrow stromal cells (BMSCs), and bone marrow dendritic cells (BM-DCs) by flow cytometry. **d** 4-1BB levels in 24-h in vitro culture supernatants of BMSCs from 6- and 18-month-old mice. **e** 4-1BB protein expression in BMSCs from 6- and 18-month-old mice was determined by western blot. β-Actin was used as an internal control for western blotting. All the data were obtained from three independent experiments. Data are shown as the means ± SEMs. *p < 0.05, **p < 0.01, ***p < 0.001, 18 mo vs. 6 mo.

Previous studies have shown that multiple cytokines and chemokines in the bone marrow microenvironment regulate the process of bone remodeling^21^. To elucidate the mechanisms underlying bone loss in aged mice, we collected the bone marrow aspirates of 6- and 18-month-old mice and examined multiple important cytokines and chemokines. A cytokine array containing specific antibodies for 24 inflammatory cytokines and chemokines was used. The results showed that there were significant changes in the levels of 10 cytokines and chemokines in the bone marrow microenvironment between 6- and 18-month-old mice (data not shown). Among these cytokines, there was a 52% increase in the 4-1BBL level in the bone marrow microenvironment of 18-month-old mice compared with 6-month-old mice (Fig. 2a). Further, 4-1BB levels in the bone marrow microenvironment were measured. The results from ELISA indicated that, like 4-1BBL, there was an 87% increase in the 4-1BB level in the bone marrow microenvironment of 18-month-old mice compared with 6-month-old mice (Fig. 2b).

To uncover the source of 4-1BBL and 4-1BB in mouse bone marrow, various types of cells within bone marrow, including HSCs, MPs, BMSCs, and BM-DCs, were isolated and subjected to flow cytometry to study their 4-1BB and 4-1BBL expression. There was no 4-1BB expression in HSCs and MPs from both 6- and 18-month-old mice (Fig. 2c). 4-1BB expression was observed in BMSCs and BM-DCs from mice, and there was an upregulation of 4-1BB expression in these two cell types from 18-month-old mice compared with 6-month-old mice (Fig. 2c). 4-1BBL expression was observed in HSCs and MPs from mice, and there was an upregulation of 4-1BBL expression in these two cell types from 18-month-old mice compared with 6-month-old mice (Fig. 2c). There was no 4-1BBL expression in BMSCs from both 6- and 18-month-old mice (Fig. 2c). Weak expression of 4-1BBL was observed in BM-DCs. However, there was no difference in 4-1BBL expression in BM-DCs between 6- and 18-month-old mice (Fig. 2c).

Considering the important role of BMSCs in bone marrow microenvironment homeostasis and bone formation^22^, we evaluated the 4-1BB levels in BMSCs. BMSCs were isolated from bone marrow of 6- and 18-month-old mice and cultured in vitro. Twenty-four-hour culture medium was collected. ELISA results showed a 74% increase in 4-1BB secretion levels in the culture medium of BMSCs from 18-month-old mice compared with 6-month-old mice (Fig. 2d). Western blotting results also showed significant upregulation of 4-1BB in BMSCs from 18-month-old mice compared with 6-month-old mice (Fig. 2e).

### Negative correlation between serum 4-1BB and 4-1BBL levels and femur trabecular bone mass in mice

**Fig. 3:**
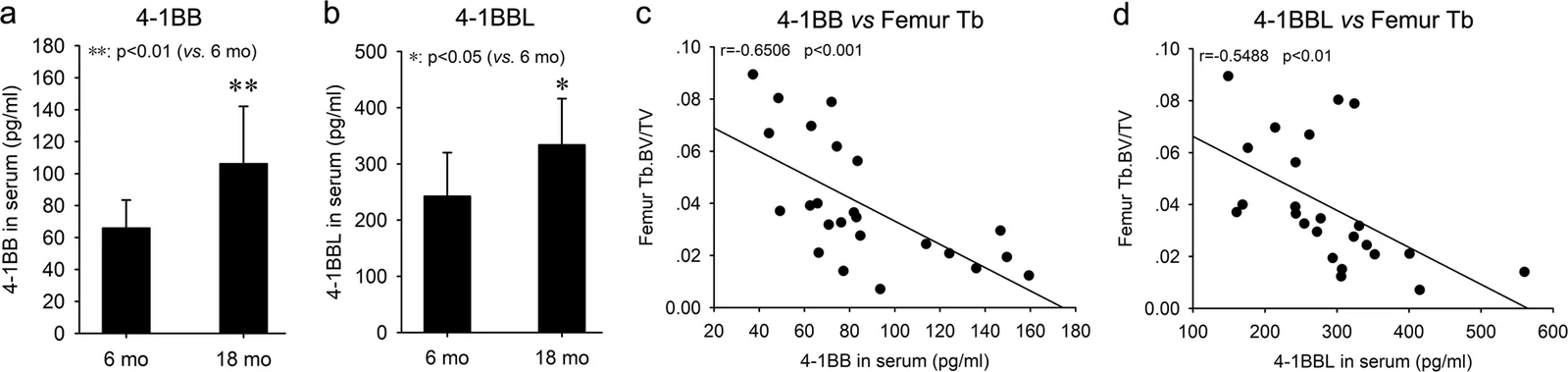
Negative correlation between serum 4-1BB and 4-1BBL levels and femur trabecular bone mass in mice. **a** 4-1BB levels in peripheral blood serum of 6- and 18-month-old mice were determined by ELISA. **b** 4-1BBL levels in peripheral blood serum of 6- and 18-month-old mice. **c** Correlation analysis between serum 4-1BB levels and BV/TV of the distal femur Tb of mice 6–18 months of age. **d** Correlation analysis between serum 4-1BBL levels and BV/TV of the distal femur Tb of mice 6–18 months of age. All the data were obtained from three independent experiments. Data are shown as the means ± SEMs. *p < 0.05, **p < 0.01, ***p < 0.001, 18 mo vs. 6 mo.

There is a close relationship between bone marrow and peripheral blood^23^. Therefore, 4-1BB and 4-1BBL levels in peripheral blood serum were studied by ELISA. There were a 61% increase in 4-1BB (Fig. 3a) and a 38% increase in 4-1BBL (Fig. 3b) levels in serum from 18-month-old mice compared with 6-month-old mice.

**Fig. 4:**
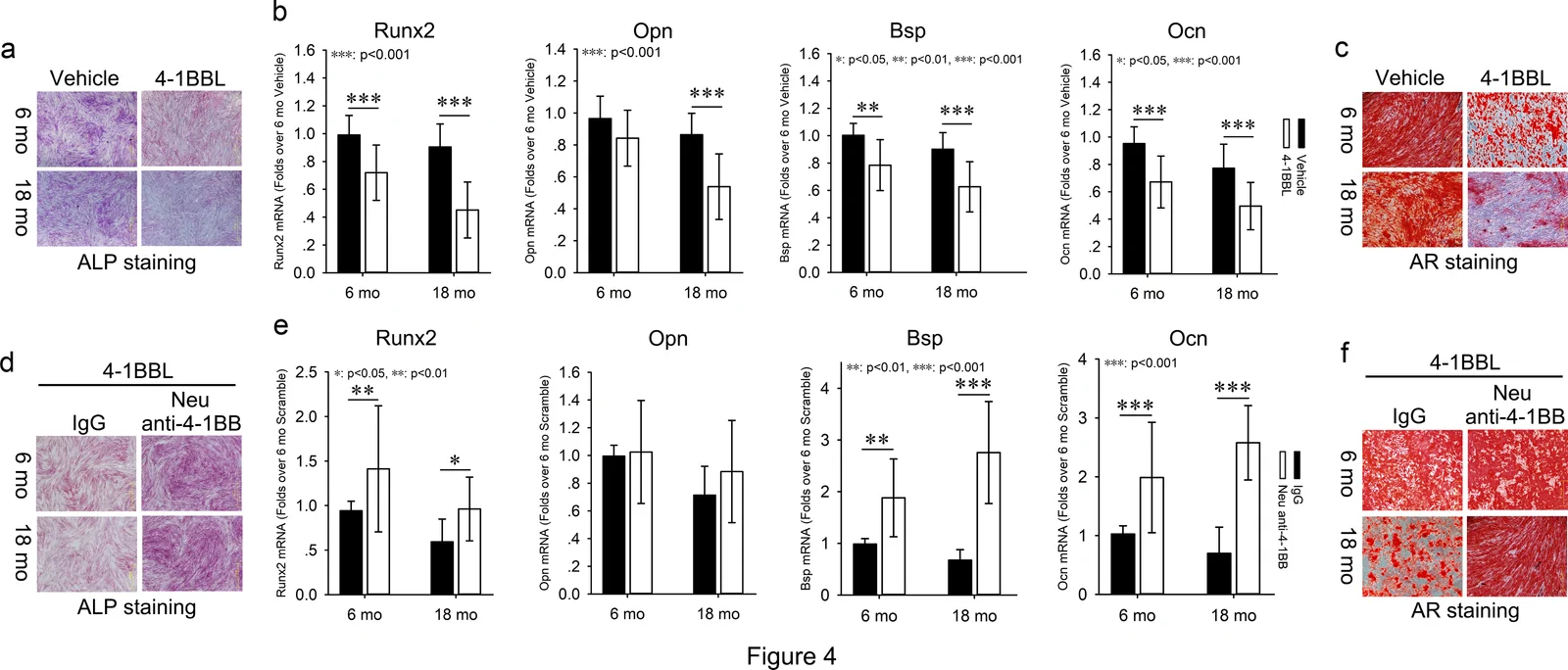
Involvement of 4-1BB upregulation in the defective osteogenic differentiation potential of BMSCs from aged mice. **a** ALP staining of BMSCs at week 1 after osteogenic induction with and without 4-1BBL treatment. **b** mRNA expression of osteogenic differentiation markers including Runx2, Opn, Bsp, and Ocn in BMSCs with and without 4-1BBL treatment was determined by qPCR. **c** Alizarin red staining of BMSCs at week 4 after osteogenic induction with and without 4-1BBL treatment. **d** ALP staining of BMSCs at week 1 after osteogenic induction with and without neutralization antibody against 4-1BB. **e** mRNA expression of osteogenic differentiation markers including Runx2, Opn, Bsp, and Ocn in BMSCs with and without neutralization antibody against 4-1BB. **f** Alizarin red staining of BMSCs at week 4 after osteogenic induction with and without neutralization antibody against 4-1BB. β-Actin was used as an internal control in qPCR. All the data were obtained from three independent experiments. Data are shown as the means ± SEMs. *p < 0.05, **p < 0.01, ***p < 0.001.

Having observed bone loss and an increase in serum 4-1BB and 4-1BBL levels in aged mice, we asked whether they were correlated. To answer this question, we explored the correlation analysis of serum 4-1BB and 4-1BBL levels with trabecular bone BV/TV of the femur, tibia, and L1 vertebra of mice within 6–18 months age. The results showed that the femur trabecular bone BV/TV was negatively associated with serum 4-1BB (*r* = −0.6506, *p* < 0.001, Fig. 3c) and 4-1BBL (*r* = −0.5488, *p* < 0.01, Fig. 3d). As with the femur, negative correlations of serum 4-1BB and 4-1BBL levels with the tibia and vertebrae BV/TV were also observed (Fig. S1).

### Involvement of 4-1BB upregulation in the defective osteogenic differentiation potential of BMSCs from aged mice

BMSCs are responsible for bone formation^24^. In the current study, BMSCs were isolated from 6- and 18-month-old mice, cultured in vitro and induced to undergo osteogenic differentiation. Some osteogenic markers including ALP, Runx2, Opn, Bsp, and Ocn and mineral deposition were employed to monitor the osteogenic differentiation process. The results from ALP staining (Fig. 4a), qRT-PCR (Fig. 4b), and alizarin red staining (Fig. 4c) showed that the osteogenic differentiation potential of BMSCs from 18-month-old mice was mildly weaker than that of BMSCs from 6-month-old mice, although the differences were not statistically significant.

As described above, 4-1BB was upregulated in BMSCs from 18-month-old mice compared with 6-month-old mice. Likewise, 4-1BBL was more abundant in bone marrow of 18-month-old mice than 6-month-old mice. 4-1BB signaling activation requires the binding of 4-1BBL to 4-1BB^25^. To mimic the bone marrow microenvironment surrounding BMSCs and evaluate the role of 4-1BB signaling in BMSC osteogenic differentiation, cross-linked 4-1BBL was added to the culture media when BMSCs were induced to undergo osteogenic differentiation. The results from ALP staining (Fig. 4a), qRT-PCR (Fig. 4b), and alizarin red staining (Fig. 4c) showed that, following 4-1BB signaling activation by 4-1BBL, the osteogenic differentiation potential of BMSCs from 6- and 18-month-old mice was significantly inhibited. It should be noted that the osteogenic differentiation potential of BMSCs from 18-month-old mice was significantly weaker than that of BMSCs from 6-month-old mice.

Neutralization antibody against 4-1BB was added to culture media to block 4-1BB signaling activation by 4-1BBL when BMSCs were induced to undergo osteogenic differentiation. The results from ALP staining (Fig. 4d), qRT-PCR (Fig. 4e), and alizarin red staining (Fig. 4f) showed that the osteogenic differentiation potential of BMSCs from 6- and 18-month-old mice was rescued in response to the blockade of 4-1BB signaling.

### DKK1 upregulation by 4-1BB via p38 MAPK in BMSCs of aged mice

**Fig. 5:**
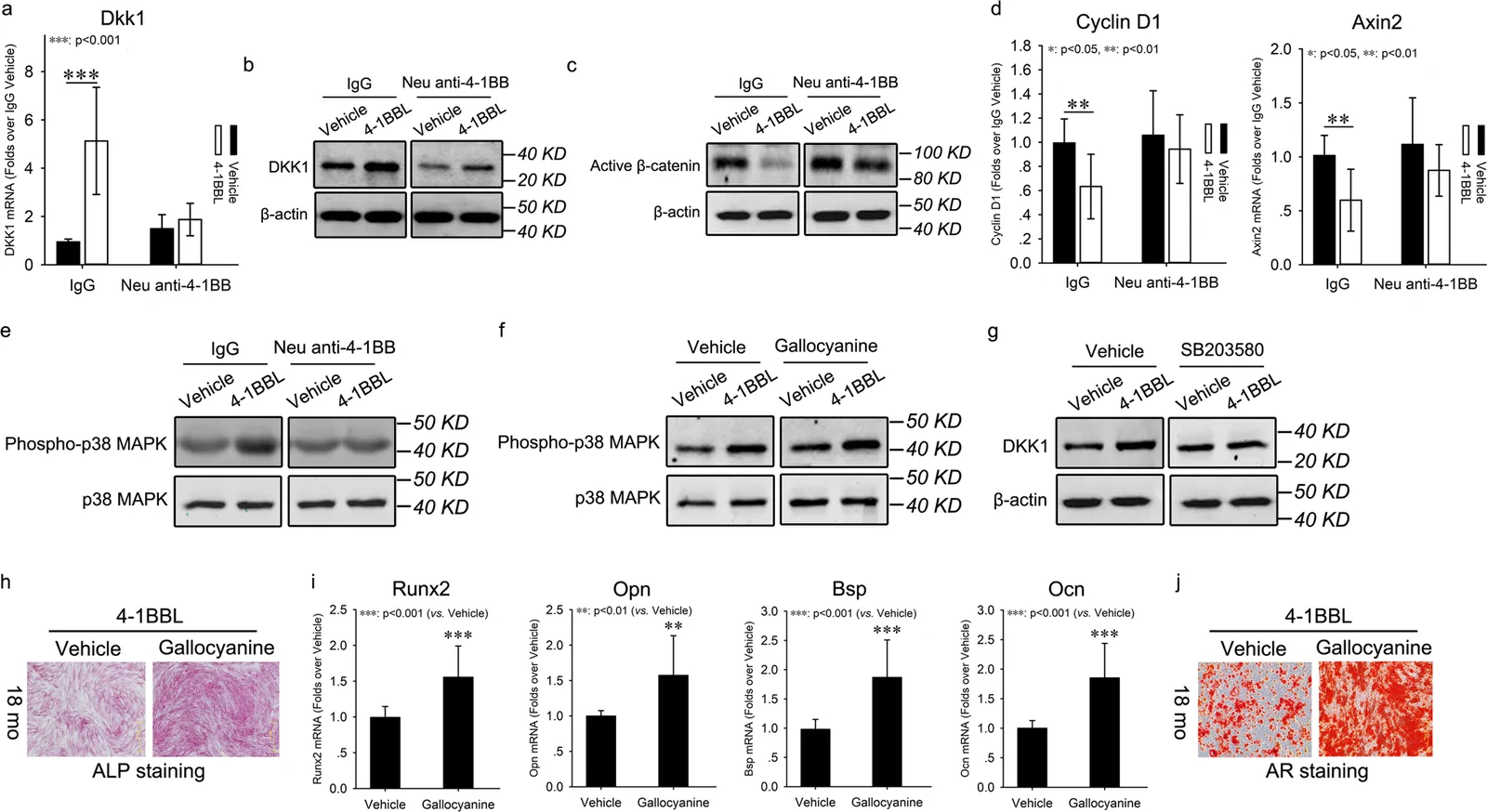
DKK1 upregulation by 4-1BB via p38 MAPK in BMSCs from aged mice. **a** DKK1 mRNA expression in BMSCs from 18-month-old mice in response to 4-1BBL and/or neutralization antibody against 4-1BB for 12 h was determined by qPCR. **b** DKK1 protein expression in BMSCs from 18-month-old mice in response to 4-1BBL and/or neutralization antibody against 4-1BB for 12 h was determined by western blot. **c** Active β-catenin protein expression in BMSCs from 18-month-old mice in response to 4-1BBL and/or neutralization antibody against 4-1BB for 12 h. **d** mRNA expression of Cyclin D1 and Axin2 in BMSCs from 18-month-old mice in response to 4-1BBL and/or neutralization antibody against 4-1BB for 12 h. **e** Phospho-p38 MAPK protein expression in BMSCs from 18-month-old mice in response to 4-1BBL and/or neutralization antibody against 4-1BB for 6 h. **f** Phospho-p38 MAPK protein expression in BMSCs from 18-month-old mice in response to 4-1BBL and/or gallocyanine treatment for 6 h. **g** DKK1 protein expression in BMSCs from 18-month-old mice in response to 4-1BBL and/or SB203580 treatment for 12 h. **h** ALP staining of BMSCs at week 1 after osteogenic induction with and without gallocyanine treatment. **i** mRNA expression of osteogenic differentiation markers including Runx2, Opn, Bsp, and Ocn in BMSCs with and without gallocyanine treatment. **j** Alizarin red staining of BMSCs at week 4 after osteogenic induction with and without gallocyanine treatment. All the data were obtained from three independent experiments. Data are shown as the means ± SEMs. *p < 0.05, **p < 0.01, ***p < 0.001.

To elucidate the molecular mechanisms underlying the 4-1BB-mediated defective osteogenic differentiation potential of BMSCs from aged mice, we investigated the responses of 4 essential signaling pathways controlling BMSC osteogenic differentiation, including TGFβ^26^, Wnt^27^, Notch^28^, and Hedgehog^29^, to 4-1BB signaling activation. Of these, the Wnt/β-catenin signaling pathway exhibited profound changes. As an inhibitor of Wnt/β-catenin signaling pathway, Dkk1 mRNA (Fig. 5a) and protein (Fig. 5b) levels were significantly upregulated in response to 4-1BB signaling activation in BMSCs from aged mice. When neutralization antibody against 4-1BB was used to block 4-1BBL-mediated 4-1BB signaling activation, the upregulation of Dkk1 mRNA (Fig. 5a) and protein (Fig. 5b) was prevented, suggesting that 4-1BB signaling activation triggers Dkk1 upregulation.

Dkk1 dampens Wnt signaling by forming a tertiary complex with LRP5/6 and Kremen-1, thereby promoting internalization of the receptor complex and β-catenin proteasomal degradation^27^. In the current study, in response to 4-1BB-triggered Dkk1 upregulation, the downregulation of active (non-phospho) β-catenin (Fig. 5c) and Wnt target genes including cyclin D1 and axin2 (Fig. 5d) was observed in BMSCs of 18-month-old mice. When neutralization antibody against 4-1BB blocked 4-1BB signaling, the downregulation of activate β-catenin (Fig. 5c) and Wnt target genes (Fig. 5d) was rescued.

To elucidate the mechanisms underlying Dkk1 upregulation by 4-1BB signaling activation, we focused on p38 MAPK, which has been shown to be a downstream effector of 4-1BB in a previous study^30^. In the current study, p38 MAPK was phosphorylated in response to 4-1BB signaling activation in BMSCs from 18-month-old mice (Fig. 5e), which was prevented by neutralization antibody against 4-1BB (Fig. 5e). Then, the specific inhibitor of Dkk1, gallocyanine, was employed to block Dkk1. The results from western blotting showed that blocking Dkk1 failed to change 4-1BB-mediated p38 MAPK phosphorylation (Fig. 5f). In contrast, when p38 MAPK was inhibited by SB203580, 4-1BB-mediated Dkk1 upregulation was blocked in BMSCs from aged mice (Fig. 5g), suggesting that 4-1BB could upregulate Dkk1 expression via p38 MAPK.

To confirm the role of Dkk1 in the 4-1BB-mediated defective osteogenic potential of BMSCs, we added gallocyanine to the culture media to block Dkk1 activation by 4-1BB/4-1BBL signaling when BMSCs were induced to undergo osteogenic differentiation. The results from ALP staining (Fig. 5h), qRT-PCR (Fig. 5i), and alizarin red staining (Fig. 5j) showed that the 4-1BB-mediated defective osteogenic differentiation potential of BMSCs from 18-month-old mice was rescued in response to the blockade of Dkk1.

### Rescue of trabecular bone loss in aged mice through in vivo blocking of 4-1BB signaling

**Fig. 6:**
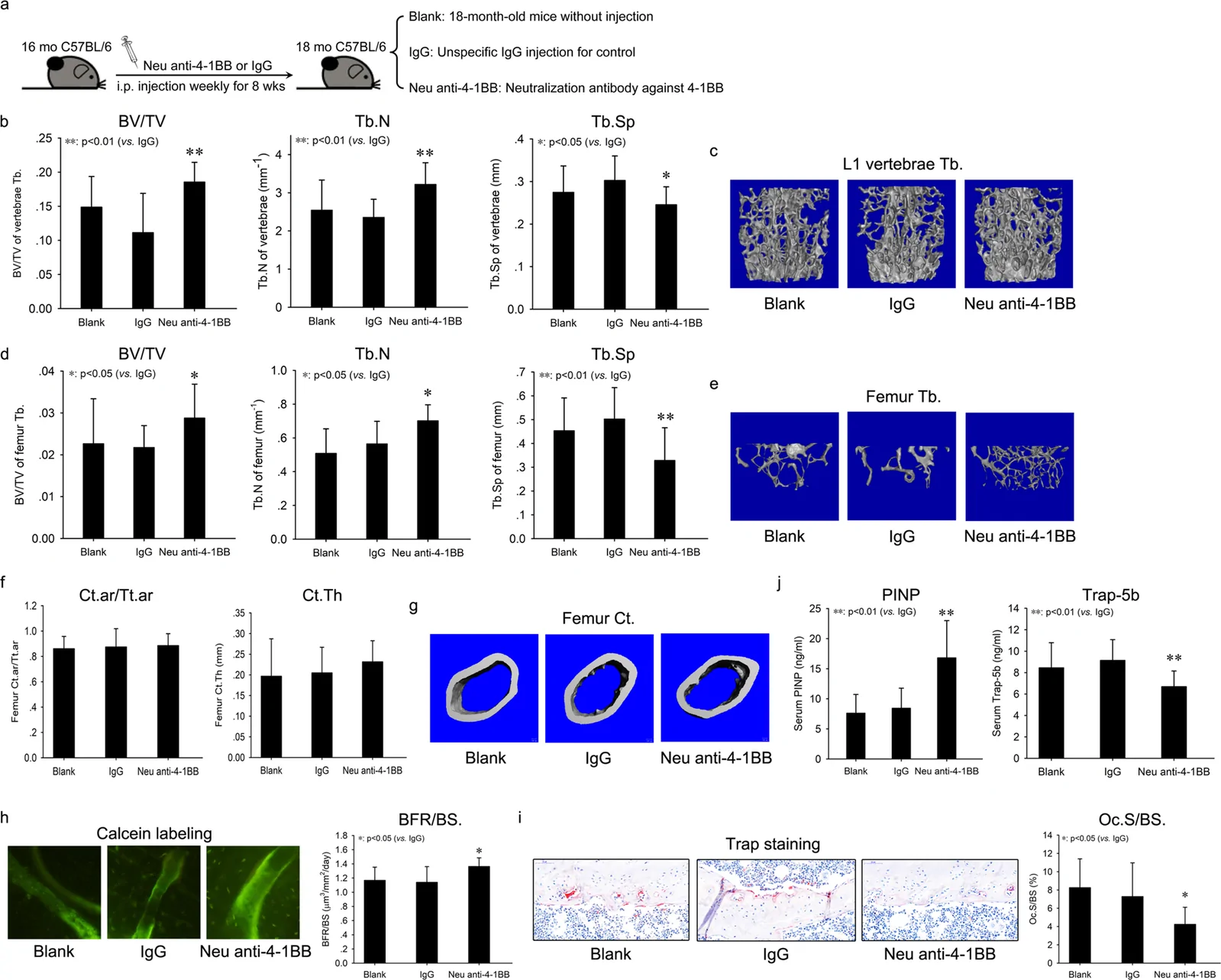
Rescue of trabecular bone loss in aged mice through in vivo blockade of 4-1BB signaling. **a** Experimental procedure. **b** BV/TV, Tb.N, and Tb.Sp of L1 vertebrae from 18-month-old mice with and without neutralization antibody against 4-1BB were determined by micro-CT. **c** Representative 3D reconstruction images of the L1 vertebrae Tb. **d** BV/TV, Tb.N, and Tb.Sp of the distal femur. **e** Representative 3D reconstruction images of the distal femur Tb. **f** Ct.ar/Tt.ar and Ct.Th of cortical bone in the femur mid-shaft. **g** Representative 3D reconstruction images of femur Ct. **h** Double calcein labeling images and bone formation rate (BFR) quantification of 18-month-old mice with and without neutralization antibody against 4-1BB. **i** Trap staining images and Oc.S/BS quantification of 18-month-old mice with and without neutralization antibody against 4-1BB. **j** Serum levels of the bone turnover markers PINP and Trap-5b from 18-month-old mice with and without neutralization antibody against 4-1BB were determined by ELISA. β-Actin was used as an internal control in qPCR. All the data were obtained from three independent experiments. Data are shown as the means ± SEMs. *p < 0.05, **p < 0.01, ***p < 0.001.

Sixteen-month-old mice were intraperitoneally administered neutralization antibody against 4-1BB weekly (Fig. 6a). After 8 weeks, animals were euthanatized for micro-CT scanning.

There was a 67% increase in BV/TV, a 37% increase in Tb.N and a 19% decrease in Tb.Sp in the L1 vertebrae of mice administered neutralization antibody against 4-1BB compared with IgG control (Fig. 6b). There was a 33% increase in BV/TV, a 24% increase in Tb.N and a 35% decrease in Tb.Sp in the distal femur of mice administered neutralization antibody against 4-1BB compared with IgG control (Fig. 6d). However, no improvements in cortical bone were observed in response to neutralization antibody against 4-1BB compared with IgG control (Fig. 6f). Micro-CT 3D reconstruction images also showed the same pattern (Fig. 6c, e and g).

In double calcein labeling assays, a 20% increase in the BFR was observed in 18-month-old mice in response to the neutralization antibody against 4-1BB compared with IgG control (Fig. 6h). In histomorphometric analysis of Trap^+^ osteoclasts, a 42% decrease in Oc.S/BS was observed in 18-month-old mice in response to neutralization antibody against 4-1BB compared with IgG control (Fig. 6i).

Accordingly, a significant increase in PINP was observed in the serum from mice administered neutralization antibody against 4-1BB compared with IgG control (Fig. 6j). In addition, there was a significant decrease in the Trap-5b level in the serum from mice administered neutralization antibody against 4-1BB compared with IgG control (Fig. 6j).

## Discussion

The main objective of this study was to elucidate the role of 4-1BB signaling in bone loss of aged mice. Generally, the rapid growth of young mice is accompanied by significant increases in body mass, bone size, mineral mass, mechanical properties, and bone formation indices. Bone length, mass, and mechanical properties reach mature levels by 18–24 weeks of age. After 42 weeks, age-related osteopenia was observed, as represented by decreased bone mass and mineralization, diminished whole bone stiffness and energy to fracture, increased size of the medullary cavity, decreased cortical thickness, decreased rate of new bone formation and the dramatic appearance of large intracortical resorption cavities. In the current study, 6-month-old mice were defined as “young” mice, and 18-month-old mice were defined as “aged” mice. The results from micro-CT, double calcein labeling, and histomorphometric analysis of osteoclasts indicated significant loss of cortical and trabecular bone resulting from decreased new bone formation and increased bone resorption in 18-month-old mice compared with 6-month-old mice (Fig. 1), which was comparable with the bone phenotypes of aged mice described above.

Osteoblasts responsible for new osteoid synthesis are derived from BMSC osteogenic differentiation. Therefore, to elucidate the mechanisms underlying the trabecular bone loss of aged mice, we focused on defective BMSC osteogenesis in aged mice in the present study. BMSCs are specifically located in the bone marrow microenvironment that is responsible for their complicated functions^7,8^. BMSC osteogenic differentiation is triggered by a variety of external stimuli from the bone marrow microenvironment. Analyzing the differences in the bone marrow microenvironment between young and aged mice is of key importance for elucidating the molecular mechanisms underlying defective bone formation.

With aging, senescence-associated molecules are secreted into the bone marrow microenvironment by senescent cells^31,32^. These molecules dampen osteogenic differentiation as well as promote BMSC senescence, which is considered to be the primary cause of osteoporotic bone loss^33,34^. Importantly, these molecules could potentially transmit signals within the bone marrow microenvironment, thereby regulating the status of bone remodeling. The present study found the accumulation of 4-1BBL and 4-1BB in the bone marrow of aged mice compared with young mice (Fig. 2a, b). Further, HSCs, MPs, BMSCs, and BM-DCs were isolated, and 4-1BB and 4-1BBL expression was investigated in these cells. 4-1BB expression was observed in BMSCs and BM-DCs. Some previous reports demonstrating 4-1BB expression in BM-DCs^35,36^ supported our current study. To the best of our knowledge, the current study is the first to show 4-1BB expression in BMSCs. For 4-1BBL, its expression was observed in HSCs, MPs, and BM-DCs, although there was weak expression of 4-1BBL in BM-DCs, which is supported by previous studies^35–37^. More importantly, significant upregulation of 4-1BB expression in BMSCs and BM-DCs and 4-1BBL expression in HSCs and MPs were observed in bone marrow from 18-month-old mice compared with 6-month-old mice. Thus, we conclude that the upregulation of 4-1BB expression in BMSCs and BM-DCs and the upregulation of 4-1BBL expression in HSCs and MPs contribute to 4-1BB and 4-1BBL accumulation in bone marrow from 18-month-old mice, at least in large part, if not completely.

As is the case for most of the TNFRSF members, 4-1BB exists in a membrane-bound and a soluble form. The soluble form of 4-1BB (s4-1BB) is produced by differential splicing. Functionally, s4-1BB binds to 4-1BBL and abrogates ligand-mediated activities such as T cell proliferation and IL-2 secretion^38,39^. 4-1BBL is a transmembrane polypeptide that belongs to the TNF ligand superfamily. Just as for 4-1BB, 4-1BBL also exists in a membrane-bound and a soluble form. In several previous studies, serum samples from patients with RA contain elevated levels of soluble 4-1BB and 4-1BBL, which correlate with disease severity^40,41^. Therefore, in addition to bone marrow, 4-1BB and 4-1BBL secretion levels were investigated in the peripheral blood serum of 6- and 18-month-old mice. 4-1BB and 4-1BBL secretion levels were enhanced in the serum of 18-month-old mice compared with 6-month-old mice (Fig. 3a, b). Interesting data in the present study were obtained from the correlation analysis between serum 4-1BB and 4-1BBL levels and trabecular bone BV/TV. The results showed negative correlations between them (Fig. 3c, d). At present, osteoporosis is mainly diagnosed based on complaints of back pain, radiographic changes in bone, and bone mineral density at both the femur and lumbar spine^42^. However, radiographic alterations including bone loss are usually signs of mid- to late-stage osteoporosis^43^. Therefore, biochemical markers involved in increased bone turnover have been proposed as potential indicators of the degree of severity of bone loss^44^. Accumulating data support markers that represent bone turnover as being correlated with osteoporosis progression. The data of the current study suggested 4-1BB and 4-1BBL as potential biomarkers for revealing bone loss, diagnosing early osteoporosis, and monitoring disease progression.

The most important findings of the present study are the 4-1BB upregulation on aged BMSCs and the involvement of the 4-1BB/p38 MAPK/Dkk1 axis in the defective osteogenic differentiation potential of aged BMSCs. Having observed significant trabecular bone loss in aged mice, we speculated that the in vitro osteogenic differentiation potential of aged BMSCs should be much weaker than that of young BMSCs. However, the results from in vitro osteoinduction assays failed to show statistically significant differences in the osteogenic differentiation potential between aged and young BMSCs, although mild attenuation could be observed in aged BMSCs (Fig. 4). In our opinion, the main cause is that the in vitro culture medium could not completely mimic the in vivo bone marrow microenvironment surrounding BMSCs. Specifically, there is no abundance of 4-1BBL in the culture medium, which was observed to be upregulated in the bone marrow of aged mice in the current study. Therefore, 4-1BBL was added to the culture medium to activate 4-1BB signaling in BMSCs. We found that the in vitro osteogenic differentiation potential of BMSCs from both 6- and 18-month-old mice was inhibited significantly in response to 4-1BB signaling activation. Moreover, the decline in aged BMSCs was more remarkable than that of young BMSCs, which is very likely to result from the upregulation of 4-1BB on aged BMSCs compared with young BMSCs. On the basis of these data, neutralization antibody against 4-1BB was employed to block 4-1BB in BMSCs following 4-1BB signaling activation by 4-1BBL. In response to this treatment, the osteogenic differentiation potential was enhanced in BMSCs from both 6- and 18-month-old mice. These data suggested that 4-1BB upregulation in BMSCs played an important role in the defective osteogenesis of aged BMSCs at least in large part, if not in whole.

A number of intracellular molecules involved in various signaling pathways are triggered when the 4-1BB/4-1BBL system is activated. Similar to the signaling pathways that are activated by other members of the TNFRSF, 4-1BB triggers activation of TRAF (TNF receptor-associated factor) 1, TRAF2, and TRAF3. TRAF2 signaling is required to activate JNK/SAPK as a result of ASK-1 stimulation^45^. In addition, NF-κB and a number of protein tyrosine kinases downstream including p38 MAPK, MEK, ERK1/2, and PI3K are induced^46^. In the current study, we found that 4-1BB could upregulate Dkk1 expression via activation of p38 MAPK in aged BMSCs. Dkk1 upregulation led to the degradation of β-catenin and further inhibited BMSC osteogenic differentiation. We believe that the 4-1BB/p38 MAPK/Dkk1 axis contributes to the defective osteogenic differentiation potential of aged BMSCs.

In the current study, we attempted to evaluate the application of neutralization antibody against 4-1BB in the treatment of trabecular bone loss of aged mice. The result showed that trabecular bone loss in aged mice could be rescued in response to blocking 4-1BB in vivo. In fact, intensive efforts have been made to treat inflammatory diseases through TNF neutralization, and multiple TNF-blocking agents, such as adalimumab, certolizuman pegol, etanercept, golimumab, and infliximan, are now approved for diseases such as juvenile idiopathic arthritis, psoriasis, psoriatic arthritis, spondylarthritis, inflammatory bowel disease, and uveitis^47,48^. To our knowledge, we are the first to show that 4-1BB neutralization could alleviate trabecular bone loss in aged mice.

Interesting data came from the histomorphometric analysis of Trap^+^ osteoclasts indicating that there was a significant decrease in Oc.S/BS in 18-month-old mice in response to neutralization antibody against 4-1BB compared with IgG control. In parallel, the serum levels of Trap-5b, a bone resorption marker, declined in response to in vivo 4-1BB neutralization. These data imply that bone resorption activity could be inhibited by blocking 4-1BB signaling, which is supported by a previous study^16^ demonstrating that the 4-1BBL and 4-1BB expressed on osteoclast precursors promote RANKL-induced osteoclastogenesis through bidirectional signaling. 4-1BBL and 4-1BB expression on osteoclast precursors was previously reported^16–18^. A role for 4-1BB and 4-1BBL in osteoclastogenesis is highlighted by several lines of evidence. First, bone marrow monocytes (BMMs) from 4-1BB^−/−^ mice exhibited decreased osteoclastogenic ability compared with 4-1BB^+/+^ mice ex vivo. Second, immobilized r4-1BBL and r4-1BB improved the RANKL-mediated osteoclastogenesis by BMMs from 4-1BB^+/+^ mice. Third, 4-1BB^-/-^ mice exhibited increased bone mass in vivo. However, it should be noted that there are some controversial results from two other groups demonstrating the inhibition of osteoclast differentiation by 4-1BB via reverse signaling through 4-1BBL^17,18^. We attribute this discrepancy to the inhibition of the interaction between endogenous 4-1BBL and 4-1BB^17,18^. However, how the stoichiometry is determined in the interaction of r4-1BB and the endogenous ligand and receptor is not clear and needs further investigation.

**Fig. 7:**
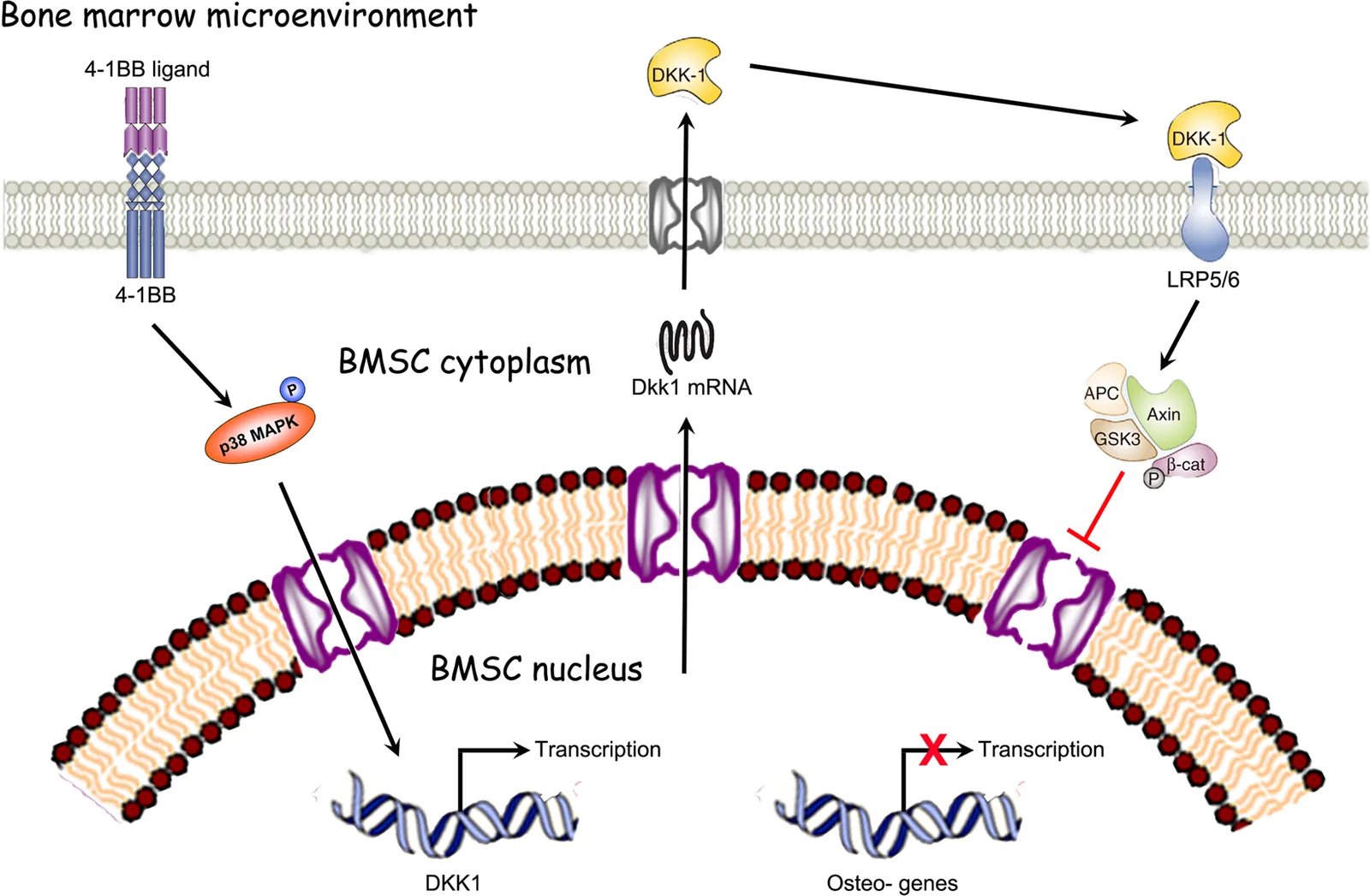
The 4-1BB-p38 MAPK-Dkk1 signaling pathway in BMSCs of aged mice. When 4-1BBL binds to 4-1BB on BMSCs from aged mice, 4-1BB signaling is activated so that p38 MAPK is phosphorylated. DKK1 expression is upregulated in response to p38 MAPK phosphorylation. DKK1 blocks Wnt/β-catenin signaling pathway and then dampens the BMSC osteogenic differentiation potential of aged mice.

In summary, the current study found the accumulation of 4-1BB and 4-1BBL in bone marrow and upregulation of 4-1BB in BMSCs from aged mice compared with young mice. In addition, 4-1BB signaling activation dampened the osteogenic differentiation potential of aged BMSCs via the p38 MAPK-Dkk1 axis (Fig. 7). In vivo 4-1BB neutralization could rescue trabecular bone loss in aged mice by increasing new bone formation and decreasing bone resorption. Our study will benefit not only our understanding of the pathogenesis of age-related bone loss but also the search for new therapy targets to treat senile osteoporosis.

## Supplementary information

supplementary data

## Data Availability

The data that support the findings of this study are openly available in Mendeley at https://doi.org/10.17632/t6byfz8r83.1.
