# Author Correction: Activation of 4-1BB signaling in bone marrow stromal cells triggers bone loss via the p-38 MAPK-DKK1 axis in aged mice

**DOI:** 10.1038/s12276-026-01875-0

**Published:** 2026-10-29

**Authors:** Daqian Wan, Songtao Ai, Huoniu Ouyang, Liming Cheng

**Affiliations:** 1 Department of Orthopedics, Tongji Hospital affiliated with Tongji University School of Medicine, Shanghai, China; 2 Key Laboratory of Spine and Spinal Cord Injury Repair and Regeneration, Ministry of Education of the People’s Republic of China, Shanghai, China; 3 Department of Radiology, Shanghai Ninth People’s Hospital, Shanghai Jiaotong University School of Medicine, Shanghai, China; 4 Department of Neurosurgery, Shanghai Ninth People’s Hospital, Shanghai Jiaotong University School of Medicine, Shanghai, China

Correction to: *Experimental & Molecular Medicine* https://doi.org/10.1038/s12276-021-00605-y, published online 15 April 2021

In this article, the wrong figure appeared as Fig. 4. The results of 18mo IgG in Fig. 4f and 18mo Gallocyanine in Fig. 5j were incorrectly reused in the AR staining panels. The results of 6mo 4-1BBL and 18mo Vehicle in Fig. 4c were incorrectly reused in the AR staining panels.

The corrected figure should have appeared as shown below.

Incorrect figure 4

**Figure:**
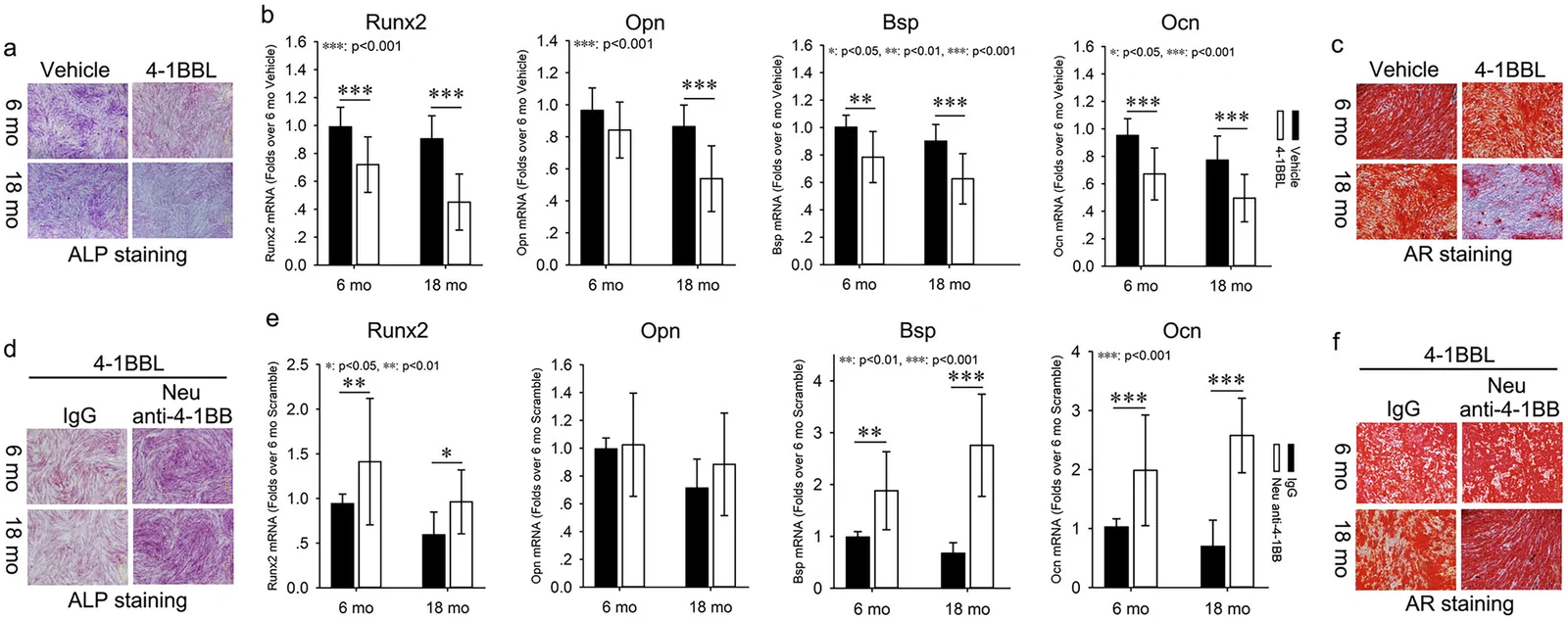

Correct figure 4

**Figure:**
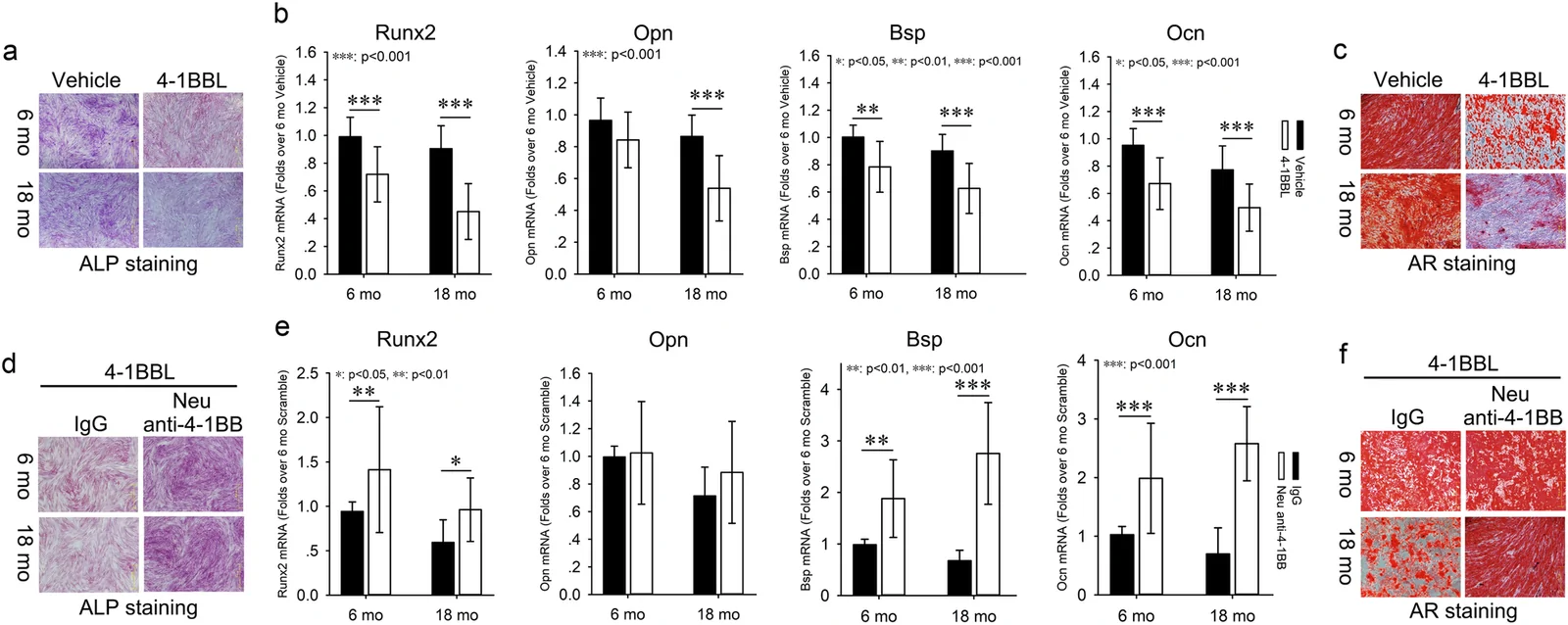

We updated the 6mo 4-1BBL panel in Fig. 4c and the 18mo IgG panel in Fig. 4f.

The authors apologize for any inconvenience caused.

The original article has been corrected.

