# Supplementary material for: Activation of 4-1BB signaling in bone marrow stromal cells triggers bone loss via the p-38 MAPK-DKK1 axis in aged mice
Source: Exp Mol Med. 2021 Apr 15;53(4):654–66. doi: 10.1038/s12276-021-00605-y (PMC8102492; doi:10.1038/s12276-021-00605-y)
Supplement: Supplementary file 1 — supplementary data [file 12276_2021_605_MOESM1_ESM.doc]

**Table S1: Primer sequences used in quantitative RT-PCR**

| **Gene** | **Primer sequences (5’-3’)** | **Product length (bp)** |
| --- | --- | --- |
| Runx2 | TTCAACGATCTGAGATTTGTGGG (F) | 221 |
|  | GGATGAGGAATGCGCCCTA (R) |  |
| Osteopontin | CCAGCAGCTCACACTGAAGA (F) | 207 |
|  | AAAAGTCTGTCGGAGTGCTGA (R) |  |
| Bsp | ATGGAGACGGCGATAGTTCC (F) | 148 |
|  | CTAGCTGTTACACCCGAGAGT (R) |  |
| Osteocalcin | GCTACCTTGGAGCCTCAGTC (F) | 71 |
|  | AGGGTTAAGCTCACACTGCT (R) |  |
| Dkk-1 | TCTCTATGAGGGCGGGAACA (F) | 156 |
|  | TTTCGGCAAGCCAGACAGAT (R) |  |
| Cyclin D1 | TCAAGTGTGACCCGGACTG (F) | 175 |
|  | ATGTCCACATCTCGCACGTC (R) |  |
| Axin2 | AGGTCCTGGCAACTCAGTAAC (F) | 164 |
|  | TCTCTTAAGTCAGCAGGGGC (R) |  |
| β-actin | GGCTGTATTCCCCTCCATCG (F) | 154 |
|  | CCAGTTGGTAACAATGCCATGT (R) |  |


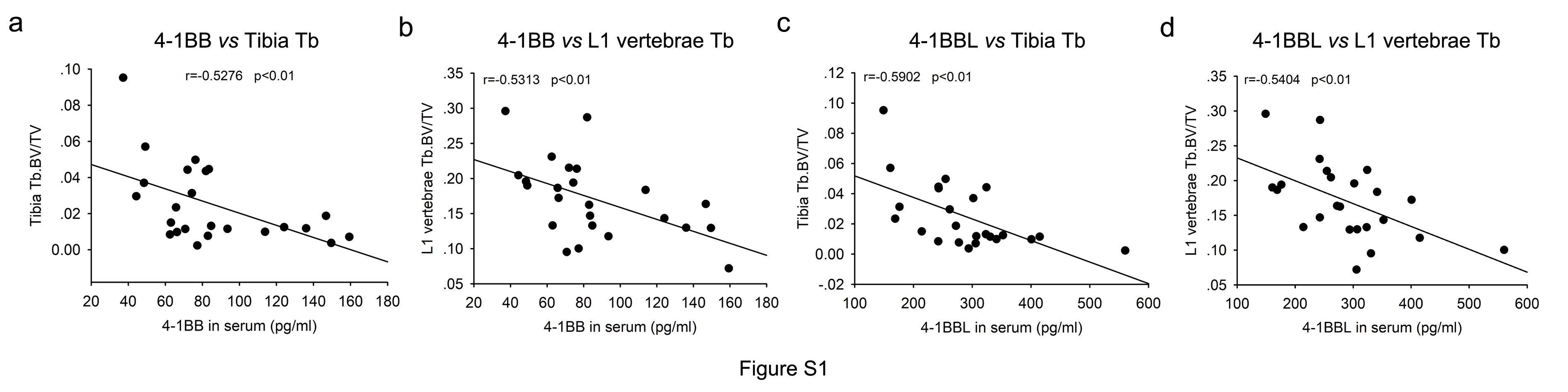


**Figure S1: Relationship of trabecular bone volume fraction of tibia and L1 vertebrae with serum 4-1BB and 4-BBL levels in mice. a:** 4-1BB vs Tibia Tb; **b:** 4-1BB vs L1 vertebrae Tb; **c:** 4-1BBL vs Tibia Tb; **d:** 4-1BBL vs L1 vertebrae Tb. Tb: trabecular bone.
